# Artificial Intelligence as a Decision-Making Tool in Forensic Dentistry: A Pilot Study with I3M

**DOI:** 10.3390/ijerph20054620

**Published:** 2023-03-06

**Authors:** Romain Bui, Régis Iozzino, Raphaël Richert, Pascal Roy, Loïc Boussel, Cheraz Tafrount, Maxime Ducret

**Affiliations:** 1 Pôle d’Odontologie, Hospices Civils de Lyon, 69008 Lyon, France; 2 Faculté d’Odontologie, Université Claude Bernard Lyon 1, Université de Lyon, 69372 Lyon, France; 3 Service de Biostatistique—Bioinformatique, Pôle Santé Publique, Hospices Civils de Lyon, 69008 Lyon, France; 4 Équipe Biostatistique-Santé, Laboratoire de Biométrie et Biologie Évolutive, UMR 5558 CNRS, Université Claude Bernard Lyon 1, Université de Lyon, 69100 Villeurbanne, France; 5 Department of Radiology, Hôpital de la Croix-Rousse, Hospices Civils de Lyon, 69004 Lyon, France; 6 CREATIS, INSA Lyon, Université Claude Bernard Lyon 1, UJM-Saint Etienne, CNRS, Inserm, UMR 5220, U1294, 69100 Villeurbanne, France; 7 Institut de Biologie et Chimie des Protéines, Laboratoire de Biologie Tissulaire et Ingénierie Thérapeutique, UMR 5305 CNRS, Université Claude Bernard Lyon 1, 69367 Lyon, France

**Keywords:** artificial intelligence, age estimation, dentistry, deep learning, machine learning, neural network, topological analysis

## Abstract

Expert determination of the third molar maturity index (I3M) constitutes one of the most common approaches for dental age estimation. This work aimed to investigate the technical feasibility of creating a decision-making tool based on I3M to support expert decision-making. Methods: The dataset consisted of 456 images from France and Uganda. Two deep learning approaches (Mask R-CNN, U-Net) were compared on mandibular radiographs, leading to a two-part instance segmentation (apical and coronal). Then, two topological data analysis approaches were compared on the inferred mask: one with a deep learning component (TDA-DL), one without (TDA). Regarding mask inference, U-Net had a better accuracy (mean intersection over union metric (mIoU)), 91.2% compared to 83.8% for Mask R-CNN. The combination of U-Net with TDA or TDA-DL to compute the I3M score revealed satisfying results in comparison with a dental forensic expert. The mean ± SD absolute error was 0.04 ± 0.03 for TDA, and 0.06 ± 0.04 for TDA-DL. The Pearson correlation coefficient of the I3M scores between the expert and a U-Net model was 0.93 when combined with TDA and 0.89 with TDA-DL. This pilot study illustrates the potential feasibility to automate an I3M solution combining a deep learning and a topological approach, with 95% accuracy in comparison with an expert.

## 1. Introduction

Decision-making is a daily process used in all medical fields including dentistry. In forensic medicine, age estimation has become an important challenge as biological markers vary notably from person to person, even in the context of identical chronological age [1]. Therefore, this context constitutes a good pilot study for the development of a decision-making procedure aiming at categorizing one individual’s age. More specifically, one of the most challenging issues involving living patients is to assess whether a person is a minor or not (i.e., under or over 18 years of age) [2]. This question is especially key in the context of unaccompanied asylum seekers without reliable documentation. The protocol of the Study Group on Forensic Age Diagnostics (AGFAD) for age estimation recommends combining a clinical examination (including the oral cavity), a hand radiograph, an orthopantomogram (OPG), and a CT scan of the clavicle medial end (if the skeletal development of the hand and wrist is completed) [3]. Following this recommendation, several techniques based on OPGs have been proposed to estimate the age of a given individual [4,5]; however, there is currently no scientific consensus regarding the best strategy.

Nearly fifteen years ago, Cameriere et al. [6] developed a topological method to estimate whether the chronological age of a given person is below or above 18 years. This approach is based on the relationship between the age and the third molar maturity index (I3M), which takes into account the apex width (a and b, respectively, for the mesial and distal apex width) and the tooth height (c) by computing the ratio: (a + b)/c [6,7]. A threshold (cut-off) value of I3M < 0.08 was identified and used to discriminate between minors and adults. This method has later been applied to several populations and has been shown to be the most efficient in discriminating between these two categories [8,9,10].

However, this technique can only be performed by a forensic expert requiring time and cost, which sometimes raises ethical issues especially in the context of unhealthy or legally implicated minors. Moreover, I3M estimation requires and depends on the experience in dental radiograph interpretation, and therefore represents a potential source of inaccuracy, as illustrated by the I3M inter-intraclass correlation coefficient (ICC) values that are good or excellent, but never perfect [11,12,13]. This difficulty in dental radiograph analysis is not specific to I3M calculation, and must therefore be carefully managed, especially given the ethical and legal impact of the final decision [14,15,16]. This dependence on human interpretation strongly highlights the need for an accurate tool able to support and standardize I3M score estimation to assist less experienced forensic experts and reduce inaccuracies.

Historically, several machine-learning-based tools were developed in dentistry. Most of them were applied to image diagnostics [17,18,19,20], while a few focused on age estimation [4,21,22,23]. These studies used either neural networks in order to directly infer the age, or the estimation of measures by an expert to perform a complex regression on the patient’s age. However, none of these studies proposed a two-stage deep learning model. This approach was able to estimate the age through an image segmentation process and a succession of post segmentation measurements, to mimic the expert’s cognitive mechanisms to compute the I3M.

Thus, the aim of the present pilot study was to investigate the technical feasibility of creating a decision-making tool based on the I3M index for forensic experts. This study presented an automated approach consisting of a mask inference on OPG followed by a topological analysis with or without a deep learning component. Performance was computed for each task, and the accuracy of their combination was finally investigated in comparison with the forensic expert’s I3M estimation. The null hypothesis was, therefore, that the I3M inferred by the solution is identical to the one estimated by the expert.

## 2. Materials and Methods

### 2.1. Study and Database

This study was conducted with accordance of the local ethical committee (HCL-21_440). A retrospective set of 530 OPGs was obtained from a previous French study [8] and thanks to an international collaboration with the Hospital of Mulago (Uganda; ethical validation SBS-216). The image collection was performed in accordance with local and national ethical standards and the Declaration of Helsinki and its later amendments. The present study was conducted and reported following as much as possible the recommendations on artificial intelligence in dentistry [24], more specifically, on the 25 items presented in the article, 23 were followed while two others (clustering and missing data management) were not applicable in our context. The OPGs were first cropped to extract left and right mandibular third molar images downsized to 256 × 256 pixels, a reasonable resolution for experts to distinguish the dental details and therefore properly assess the I3M score. Each image was analyzed by an I3M expert (CT) to assess the possibility of estimating an I3M score. Among others, radiographs without two apices or without a third mandibular molar were excluded. The final reviewed dataset was composed of 456 images, 57% of the patients were minor for an average age of 17.9 years.

### 2.2. Data Splitting, Pre-Processing, and Augmentation

A model is defined as the combination between a segmentation algorithm (mask R-CNN [25] or U-Net [26]) with one computing the I3M through a topological analysis with or without a deep learning component. In order to assess the performance of each model, 80% of the database was assigned to the training sample, and the remaining 20% to the testing sample, which was solely used to compute the statistical error once the model was trained. Before training and testing Mask R-CNN [25] and U-Net [26] (see below), a data pre-processing procedure was applied using the contrast limited adaptive histogram equalization (CLAHE) enhancement approach. This image processing enhances the contrast through a transformation function based on the neighborhood region of each pixel [27]. Data augmentation techniques were also applied using random rotations and flips of the images.

### 2.3. Labeling Process

The use of a supervised algorithm implies prior labeling on the training set. The labelling processed, performed by RB, consisted in manually creating a polygon mask (instance segmentation) of the mineralized tissue. One apical and one coronal mask were manually created using the ‘labelme’ annotation tool under the open MIT license [28].

### 2.4. Training of Mask Inference with Mask R-CNN and U-Net

Two deep learning approaches were used and compared to perform the mask inference: Mask R-CNN and U-Net.

Mask R-CNN is a region-based convolutional neural network, its backbone is the Faster-RCNN algorithm with an additional final convolutional branch enabling the prediction of an object mask [25]. Mask R-CNN is an algorithm developed by the Facebook AI Research team and was implemented, in our context, under the open MIT License (matterport) [29]. U-Net is a convolutional neural network with a characteristic U-shaped architecture that was specifically developed for biomedical image analysis and has already proven to be successful in analyzing tooth decay on X-ray images [26].

Both U-Net and Mask R-CNN are supervised learning algorithms that require labeled inputs; in our specific context, this was instance segmentation of the mineralized tissue.

In order to train the network, a batch size of 32 images was chosen for U-Net and 20 for Mask R-CNN. The weight update was performed with a stochastic gradient descent optimizer for both Mask-RCNN and U-Net. A 5-fold cross-validation approach was implemented to assess the network robustness and optimize the remaining parameters. The analysis concluded on an optimal convergence after 10 epochs for both Mask-RCNN and U-Net.

### 2.5. Topological Data Analysis without Deep Learning (TDA) or with Deep Learning (TDA-DL)

Topological Data Analysis (TDA) consists in the application of the mathematic discipline of topology to data extracted from a real system allowing the analysis of geometrical patterns such as shapes [30]. Two specific approaches were compared to compute the I3M figures based on a segmented tooth (mask).

The first step of TDA consisted of the rotation of the image by vertically aligning the barycenters of the two instances segmentations in order to compute the tooth height (parameter c). The second step consisted of applying a gradient approach to find the line equidistant to both instance segmentations. This line represented the root canal center, its ending points were the middle of the apices. The final steps consisted of finding the two extremities of each apex. Simulation of radii centered on various points of the centered line were used to partition each instance segmentation in two parts: one separating the pulp and the dentine, the other separating the tooth and the environment. The extremity of the apex constituted the transition between these two parts. Finally, the a, b, and c parameters were computed: a and b corresponded to the distance between the two extremities of each apex, and c was the distance between the lowest and the highest point on the vertical axis.

**Figure 1:**
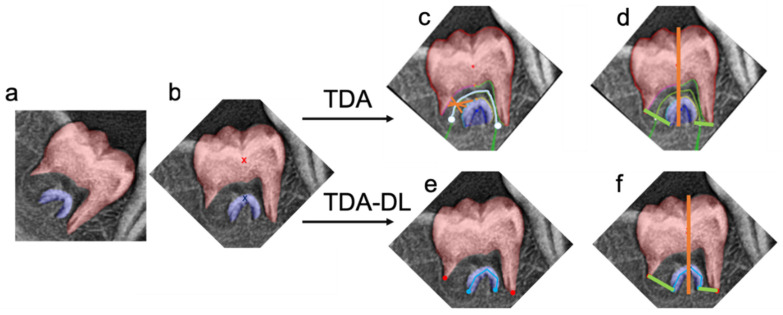
Illustration of the Topological Data Analysis (TDA) and Topological Data Analysis associated with Deep Learning (TDA-DL). Both approaches (TDA and TDA-DL) take as an input the (**a**) instance segmentation, (**b**) vertical rotated based on the barycenters (red and blue x). The TDA approach finds the (**c**) root canal middle line (cyan) based on the gradient analysis between the apical and coronal segmentation. Then, the segmentation of the root canal walls is obtained through radii analysis (orange) centered on the root canal middle line (cyan). The last points of the coronal and apical root canal walls on the left and right side of the tooth represent the ending points necessary to compute the apex diameter. (**d**) A vertical line joining the barycenters is defined in order to measure of the tooth height, more specifically this line cannot go above the most coronal point of the instance segmentation nor below the most apical point of the instance segmentation. The TDA-DL approach directly applies a deep learning approach able to automatically find the (**e**) root canal ending points (blue, red) and then (**f**) measure the tooth height and apex diameter using the obtained points.

The TDA-DL approach consisted in combining a topological data analysis followed by a deep learning approach. The first step was the rotation of the image by vertically aligning the barycenters of the two instances segmentations (apical and coronal) in order to compute the height (parameter c) of the tooth. The segmentations (apical and coronal) were then virtually separated into two sections by a vertical line joining the barycenters. The coronal limit points were defined, on the left and right side of the vertical line, as the lowest points of the coronal segmentation. In order to find the apical limit points, a deep learning approach was implemented. It consisted in fitting an inverted U-shape line at the center of the apical mask. The U-shape line was mathematically defined by five points: two ending points and three inflexion points. A U-Net convolutional network was trained to infer this apical mask skeleton. The apical limit points were then defined, on the left and right side of the vertical line, as the ending points of the inferred U-shape skeleton. The a and b parameters were then computed as the distance between the coronal and apical ending points on the left side and on the right side, respectively, of the vertical middle line (Figure 1).

### 2.6. Performance Assessment

In order to assess and understand the segmentation error, every mask inferred by the algorithm was visually checked in comparison to the expert’s radiographic graphical analysis to identify errors on the apices or on the crown.

The intersection over union metric (*IoU*) was computed to evaluate the Mask R-CNN and U-Net performance to infer the apical and coronal masks. Given *G* the ground truth mask and *I* the inferred mask, the *IoU* follows: IoU=|G∩I|/|G∪I| . In our specific context, one could label each pixel as True/False Positive/Negative, the computation of the *IoU* then becomes: IoU=TP/(TP+FN+FP). *IoU* was computed for each radiograph on the apical mask, the coronal mask, as well as on the total combined mask. Paired sample bilateral *T*-Test was computed in order to assess the existence of a statistically significant difference between maskRCNN and U-Net.

In order to properly assess the error related solely to the topological analysis, the algorithm was also applied on ground truth segmentations. The inferred metrics, a, b, and c were then compared to the expert’s ones.

In order to assess the impact of the error on the final decision, the I3M threshold was set to 0.08: for I3M < 0.08, the subject was considered as being ≥18 years old, for I3M > 0.08, the subject was considered as being <18 years old.

### 2.7. Statistical Analysis

In order to analyze the ability of the automated approach to compute I3M, a paired bilateral *T*-test analysis was performed with a type 1 error of 0.01.

The tested null hypothesis was, therefore, that the inferred I3M is equivalent to the one computed by the expert.

This approach was used to compare the I3M inferred by the complete automated solution combining the deep learning and the topological approach to the expert’s one. It was also used to compare the I3M inferred by the topological analysis on the ground truth mask to the expert’s one to isolate the error inherent to the second part of the process.

Analysis of the performance of the different algorithms regarding the final decision was performed using the McNemar’s test for nominal data and the null hypothesis was the presence of an agreement.

Pearson correlations were also computed to estimate the linear dependency between the expert’s I3M and the one computed by the various algorithms.

## 3. Results

### 3.1. Performance and Errors of Mask Inference

Paired sample *T*-test of bilateral difference demonstrated a significative difference between Mask R-CNN and U-Net for the segmented masks, the apical masks, and the overall masks. In terms of mean intersection over union, U-Net performed better than Mask R-CNN for the coronal mask (U-Net: 92.9%; Mask R-CNN: 86.1%), the apical mask (U-Net: 74.8%; Mas R-CNN: 62.1%) as well as the overall mask (U-Net: 91.2%; Mask R-CNN: 83.8%) (Table 1).

**Figure 2:**
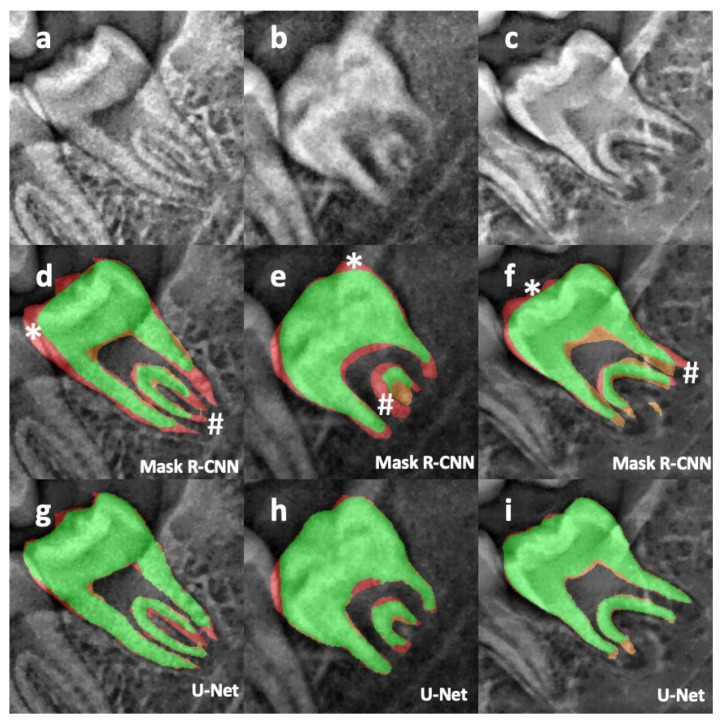
Illustration on three different radiographs (**a**–**c**) of the segmentation and its associated error with Mask-R CNN (**d**–**f**) and U-Net (**g**–**i**). Green: true positive, orange: false positive, red: false negative, #: illustration of Mask R-CNN errors on the apices, *: illustration of Mask R-CNN error on the crown.

Error analysis of the mask inference confirmed the superiority of U-Net over Mask R-CNN (Figure 2). As a matter of fact, the identification of the root apical part seemed to be more challenging for Mask R-CNN, reducing de facto the accuracy in the estimation of a + b (errors # in Figure 2d–f). It also appeared that Mask R-CNN had difficulties in identifying the crown in its entirety, which could also impact the computation of c (errors * in Figure 2d–f).

At this stage, in the training sample, approximately two thirds of the OPGs (57/88) were kept, which corresponded mostly to underage subjects (80% of the sample). Indeed, the remaining 31 OPGs presented closed or almost closed apices, which made it difficult for U-Net to identify the two different parts of the tooth, and therefore for TDA or TDA-DL to identify the two apices.

### 3.2. Performance of TDA and TDA-DL

**Figure 3:**
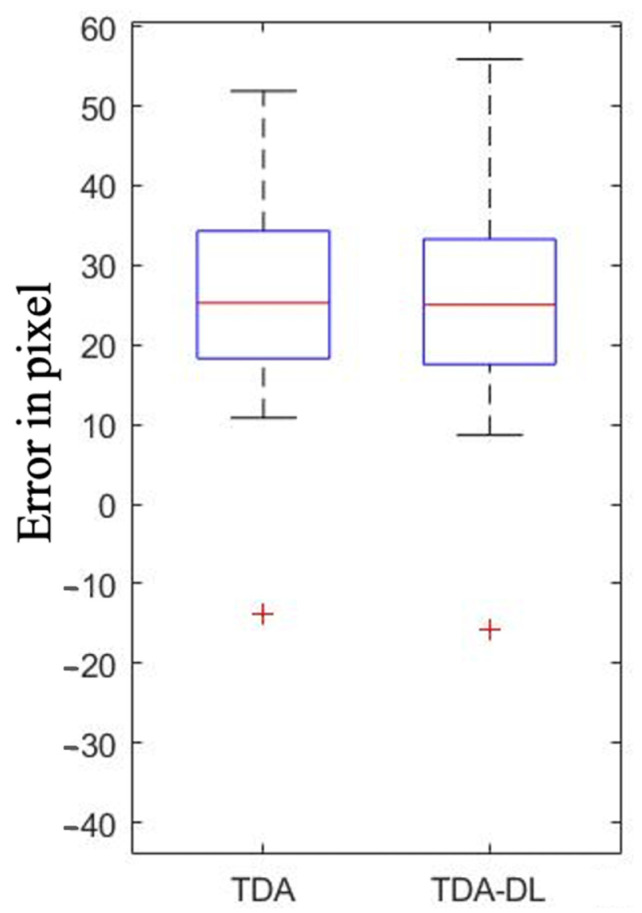
Performance of Topological Data Analysis (TDA) and Topological Data Analysis associated with Deep Learning (TDA-DL) on ground truth segmentation to determine the I3M numerator (a + b).

**Figure 4:**
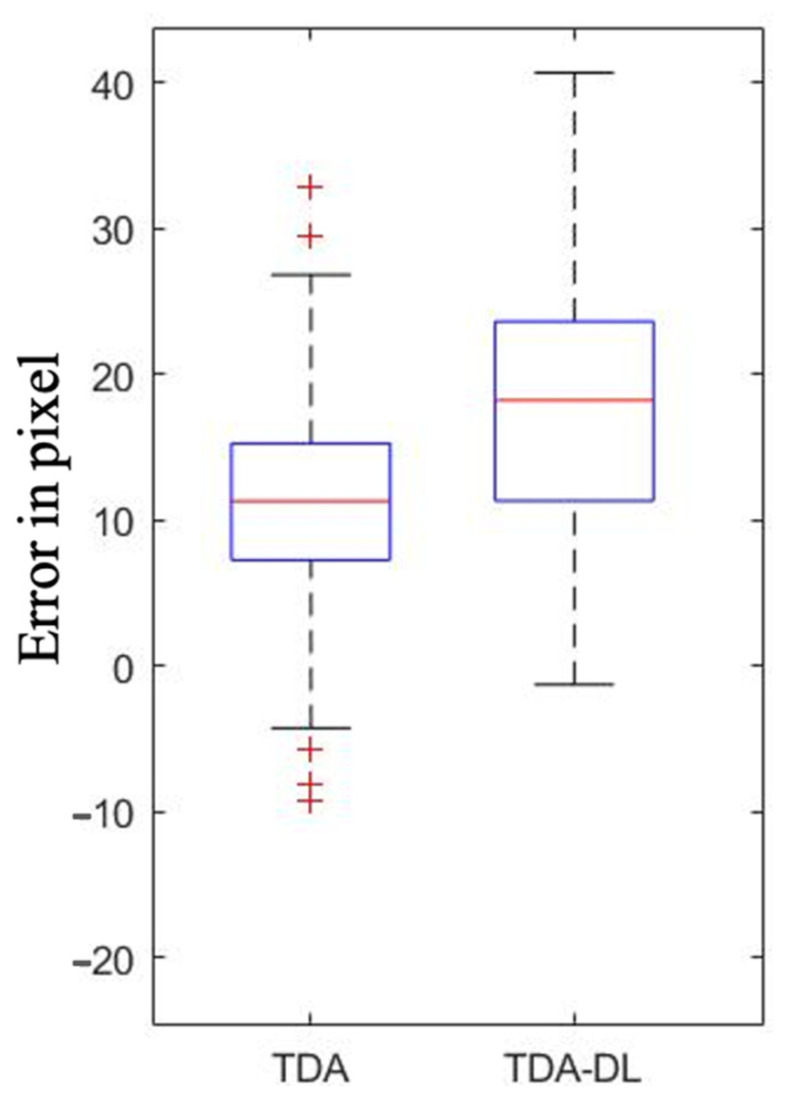
Performance of Topological Data Analysis (TDA) and Topological Data Analysis associated with Deep Learning (TDA-DL) on ground truth segmentation to determine the I3M denominator I.

Topological analysis on the ground truth mask revealed similar performance between TDA and TDA-DL. TDA was able to determine a + b with a mean absolute error (MAE) ± standard deviation (SD) of 12.26 ± 7.21 pixels in comparison with the value of the expert, while the TDA-DL MAE ± SD was 17.98 ± 9.08 pixels (Figure 3). Similarly, for c, the MAE ± SD was 26.67 ± 9.97 pixels for TDA and 26.21 ± 10.28 pixels for TDA-DL (Figure 4). Paired *T*-test analysis for TDA and TDA-DL on ground truth found *p*-values < 0.01, thereby rejecting the null hypothesis of similarity between the expert and the various combinations.

I3M’s similarity is important, however the final decision (minor or adult) remains in our context the key part. The McNemar test on agreement between the expert and the algorithm was neither rejected for TDA nor for TDA-DL, suggesting that the expert and both algorithms mainly agreed on their final decisions.

### 3.3. Performance of U-Net Combined with TDA and TDA-DL

The combined approach consisted in implementing the best segmentation algorithm followed by the different topological analyses in order to compare their ability to predict the I3M score. The MAE ± SD was 0.04 ± 0.03 for U-Net combined with TDA and 0.06 ± 0.04 for U-Net combined with TDA-DL in comparison with a dental forensic expert. Both these combinations were able to reproduce 94.7% (54/57) of the expert decisions.

Paired *T*-test analysis rejected the null hypothesis of equivalent mean only for U-Net combined with TDA (*p*-value < 0.01).

**Figure 5:**
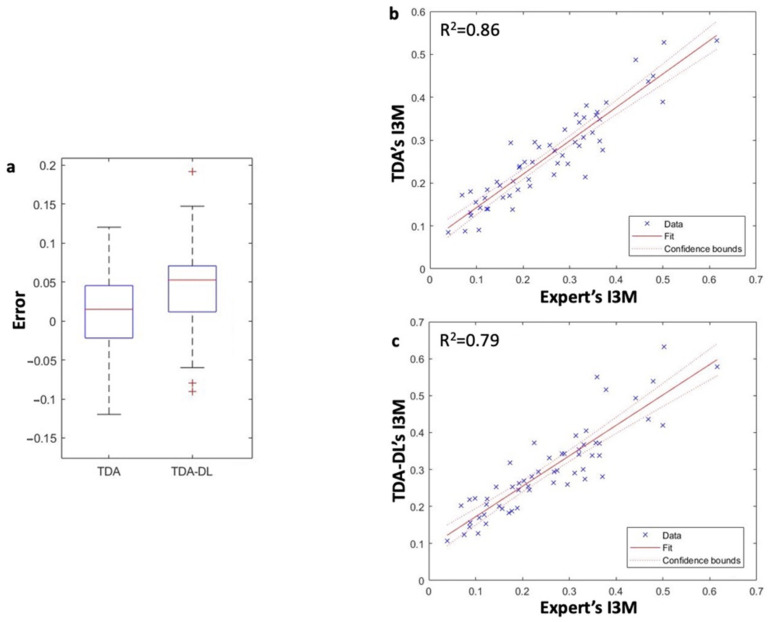
Overall comparison between TDA and TDA-DL with the expert. Performance of I3M calculation of the two solutions was analyzed in terms of error regarding I3M score (**a**). Coherence between the two solutions and the expert were analyzed through Pearson correlation (**b**,**c**).

The similarity of performance between U-Net combined with TDA or with TDA-DL was also confirmed by the analysis of the correlation between the I3M score computed by the expert and estimated by the different approaches. As a matter of fact, the Pearson correlation coefficient was of 92.77% (*p*-value < 0.01) for U-Net combined with TDA and 89.28% (*p*-value < 0.01) for U-Net combined with TDA-DL (Figure 5).

Analysis of the final decision based on the I3M score revealed a non-rejection of the McNemar test for any of the combinations considered, suggesting that the expert and algorithm combinations mainly agreed on the final decision.

## 4. Discussion

The aim of this study was to evaluate the feasibility of replicating the cognitive mechanism of a forensic expert using an innovative approach consisting in the combination of supervised deep learning approaches and automated topological algorithms. The results suggested that U-Net was the best approach to infer the mask of the mandibular third molar. They also revealed that the two topological approaches offered similar performance regarding their ability to properly infer a, b, and c, and therefore the I3M score. The developed approach was able to mimic the forensic expertise with a 94.7% accuracy. However, several methodological, technical, and ethical questions must be discussed.

Methodologically, unsupervised machine learning techniques such as deep neural network regressions have been implemented in order to prevent human errors [4,21,22,23,31]. These automated methods appear to be particularly attractive as they are fast and less time-consuming since they do not require any prior labeling work. However, these models have been reported to not be mature enough to be implemented, due to their high variance especially regarding the key chronicle age of 18 years [4]. In order to overcome such an issue, the approach of the present study consisted in being based on the I3M score, which has good results regarding the key chronicle age of 18 years [6,7]. The present procedure consisted in using an intermediate segmentation (mask). This innovative approach reduces the dimensionality of the problem by transforming the original image into simple shapes, which become a better input for the topological analysis in order to properly measure a, b, and c. Previous publications in this field have suggested segmentation with a unique mask for the whole tooth [23,31,32]. However, by definition, such approaches do not encapsulate the presence of two different tissues (mineralized tissue and pulp tissue) at the tooth apex, preventing de facto the algorithm’s ability to compute the apex width (a, b). To resolve this issue, the present study proposed the implementation of a two-mask approach. Such a strategy could easily be adapted to other dental expertise fields requiring measurements or calculus on dental radiographs, for example in order to assess endodontic complexity or to estimate bone loss [20,33,34].

Regarding the first part of the model, the algorithm produced slightly inferior results compared to previous studies attempting to segment the mandibular third molar through Mask-RCNN or U-Net [31,32,35]. This could be explained by the fact that our database included mostly young patients with starting root formation whose segmentations are more complicated due to inconsistent morphologies [31,32]. Moreover, the results are highly influenced by the apical mask, which is likely more complicated to segment due to the wide variety of anatomies and developmental stages encountered. As previously reported [35,36,37], our results revealed that U-Net was particularly indicated to infer masks on dental radiographs. Moreover, the error analysis revealed that U-Net was also better at identifying the apices of roots. This difficulty during the segmentation to identify the apices of roots entirely has already been observed for molars [38,39], and this point is particularly important for performing future accurate topological analyses.

Regarding the overall performance, U-Net combined with TDA or with TDA-DL displayed a 95% accuracy in comparison with the expert’s decision, which is promising for this first pilot study. It is important to note that these results were obtained on a database with an 80% share of underage patients given the necessity to have a large panel of open apices radiographs. Indeed, compared to other similar studies [39,40], the present study presented a relatively low number of OPGs, which might have limited the algorithm’s ability to learn all possible root configurations, and one could reasonably expect better results with a larger training database.

Graphical analysis between the expert’s measurements and the ones produced by the algorithm revealed a systematic overestimation by the latter. These discrepancies can be explained by the fact that the algorithm used the crown’s most coronal point when estimating the c value, while the expert used a reference point that is more centered on the crown; regarding a and b, the algorithm by design measured the distance between the two most apical points while the expert used points that are slightly more coronal, in other words the expert estimated a width at the apical constriction while the algorithm estimated it at the apical foramen.

This pilot study also highlighted several limitations that must be discussed. First and foremost, this study was performed on a relatively small database, even when considering the effect of data augmentation. Its results should therefore be considered with care, they are only reflecting the potential of such approach in the context of this pilot study which calls for further studies with similar approaches on a larger sample size in order to eventually consider the generalization of this solution. Moreover, the present study was performed after a manual cropping of the third molar, which limited its ease of use but also led to better results. A future perspective would be to implement an automated cropping of the mandibular third molars, such an approach appears to be relatively easy according to published studies [21,32]. It is also important to note that major differences could exist between populations, but also between OPGs themselves, thereby leading to different root configurations, positions, and radiograph qualities, potentially increasing the errors during the labeling and I3M computation [41]. Previous studies based on unsupervised approaches have offered very few explicability tools and oftentimes display direct raw results without giving the user the possibility of evaluating the intermediary step leading de facto to black box models. Notwithstanding, it is worth mentioning the Grad-CAM heat map that was proposed to improve the explicability of deep-learning-supervised solutions [4]. By reporting the masks and displaying the a, b, and c segments on the radiograph, the procedure proposed herein allows any expert or user to visually validate the mask inference as well as the computation of the score, thereby greatly improving the overall explicability and transparency. A selection bias is also inherent to the expert’s filtering process. As a matter of fact, the selection criterion was radiographic images compatible with the computation of an I3M score. In other words, only teeth with open apices were selected, leading to the creation of a subject sample for which the average age was lower than 18 years. However, further investigations could lead to the production of an algorithm able to identify the tooth stage or closed apex on a given radiograph, and therefore compensate for this bias [21,42]. Furthermore, this pilot study based itself on a single forensic expert and a relatively small database mixing radiographic images from two countries. In this context, the generalization of the proposed approach would require more extensive research on a larger, more diverse database, as well as the comparison to a panel of several I3M experts.

Finally, some ethical questions relative to the use of AI-based technologies could be raised [43], especially given the legal impact that any mistakes could have. This question is challenging; however, with respect to EU law, most of the time, the responsibility is transferred to the user (i.e., the expert) [44]. It therefore requires particular care when distributing such a technology. Critically, the aim of this study was to estimate the technical feasibility of developing a tool for educational purposes or to assist experts in uncertain cases, especially when the score averages around 0.08; this study never intended to replace forensic experts. This fully transparent approach offering visual results to the final user is particularly important since AI in dentistry is a fairly recent field of investigation, trust must be gained in order to obtain a sustained and fully accepted system [43,44,45,46].

## 5. Conclusions

This study was able to segment mandibular third molars in two parts and measure the apex width (a and b) as well as the tooth height (c). The best approach consisted in a U-Net algorithm combined with a topological approach. This study illustrated the technical feasibility of creating a decision-making tool replicating the I3M index for forensic experts with a 95% accuracy and a mean absolute error of 0.04. However, due to the numerous limitations highlighted by this proof-of-concept, further investigations are required before considering generalization and implementation of the solution.

## Figures and Tables

**Table 1:** Performance of mask inference (Mask R-CNN or U-Net) on the different segmented part of the tooth.

Segmented Mask	Mask R-CNN mIoU *	U-Net mIoU *	*p*-Value
Coronal	86.1%	92.9%	<0.01
Apical	62.1%	74.8%	<0.01
Overall	83.8%	91.2%	<0.01

* mIoU: mean intersection over union metric.

## Data Availability

Data are available by contacting the corresponding or first authors.
